# Therapeutic Benefits of Delayed Lithium Administration in the Neonatal Rat after Cerebral Hypoxia-Ischemia

**DOI:** 10.1371/journal.pone.0107192

**Published:** 2014-09-11

**Authors:** Cuicui Xie, Kai Zhou, Xiaoyang Wang, Klas Blomgren, Changlian Zhu

**Affiliations:** 1 Center for Brain Repair and Rehabilitation, Institute of Neuroscience and Physiology, University of Gothenburg, Göteborg, Sweden; 2 Department of Women's and Children's Health, Karolinska University Hospital, Stockholm, Sweden; 3 Perinatal Center, Institute of Neuroscience and Physiology, University of Gothenburg, Göteborg, Sweden; 4 Department of Pediatrics, The Third Affiliated Hospital of Zhengzhou University, Zhengzhou, China; 5 Department of Pediatrics, University of Gothenburg, The Queen Silvia Children's Hospital, Gothenburg, Sweden; 6 Department of Pediatrics, Zhengzhou Children's Hospital, Zhengzhou, China; Hôpital Robert Debré, France

## Abstract

**Aim:**

We have previously shown that lithium treatment immediately after hypoxia-ischemia (HI) in neonatal rats affords both short- and long-term neuroprotection. The aim of this study was to evaluate possible therapeutic benefits when lithium treatment was delayed 5 days, a time point when most cell death is over.

**Methods:**

Eight-day-old male rats were subjected to unilateral HI and 2 mmol/kg lithium chloride was injected intraperitoneally 5 days after the insult. Additional lithium injections of 1 mmol/kg were administered at 24 h intervals for the next 14 days. Brain injury was evaluated 12 weeks after HI. Serum cytokine measurements and behavioral analysis were performed before sacrificing the animals.

**Results:**

Brain injury, as indicated by tissue loss, was reduced by 38.7%, from 276.5±27.4 mm^3^ in the vehicle-treated group to 169.3±25.9 mm^3^ in the lithium-treated group 12 weeks after HI (p<0.01). Motor hyperactivity and anxiety-like behavior after HI were normalized by lithium treatment. Lithium treatment increased neurogenesis in the dentate gyrus as indicated by doublecortin labeling. Serum cytokine levels, including IL-1α, IL-1β, and IL-6, were still elevated as late as 5 weeks after HI, but lithium treatment normalized these cytokine levels.

**Conclusions:**

Delayed lithium treatment conferred long-term neuroprotection in neonatal rats after HI, and this opens a new avenue for future development of treatment strategies for neonatal brain injury that can be administered after the acute injury phase.

## Introduction

Perinatal asphyxia-induced brain injury remains a major cause of perinatal morbidity and mortality [1]. Severe hypoxic-ischemic brain injury occurs much more often in developing countries. Survivors of perinatal asphyxia often suffer from long-term neurological deficits and impairments such as cerebral palsy and epilepsy [2]. Perinatal brain injury involves multiple pathways that lead to both early and delayed phases of neuronal cell death [3]. Therapeutic approaches targeting these mechanisms in rodents have shown some promise [4], but the clinical relevance of these treatments is limited due to the fact that the protective agents must be administered prior to or shortly after the insult [5]. Hypothermia is currently the main therapy applied to infants who have suffered a perinatal brain injury, but its efficacy depends on the severity of the damage and how quickly hypothermia can be applied after the initial brain injury [6]. The neuroprotective effect of hypothermia is believed to occur via reduced cellular metabolism, suppression of the inflammatory cascade, decreased glutamate excitotoxicity and apoptotic cell death [7].

Active cell death peaks 24–48 h after HI and has subsided after 6 days [8], [9], but recent studies have shown that HI-induced brain injury continues to weeks after the initial acute injury phase [10]–[12]. Additional studies have reported that interfering with the delayed phase of the cell death and inflammation response (within a few days) can be neuroprotective and can reduce the severity of the initial brain injury [13]–[16]. Thus, there is an urgent need to develop new strategies to prevent both early and delayed cell death at clinically relevant time points [17].

Lithium has been used for decades to treat bipolar disease [18], [19], and it has been shown that lithium also protects neurons against excitotoxicity and apoptosis during the early phase of cell death after HI [20]. Notably, lithium increases hippocampal neurogenesis [21] and rescues memory in a mouse model of Down syndrome [22]. The therapeutic benefit of lithium has been suggested through inhibition of glycogen synthase kinase-3β (GSK-3β) [23], [24], which is critical for pro-apoptotic proteins release from mitochondria [25]. We have previously demonstrated that lithium treatment immediately after HI exerts not only impressive anti-apoptotic effects during the early phase of injury development, but also decreases inflammation and promotes neurogenesis after injury in a rat neonatal HI model [20], [21]. Thus, lithium appears to provide both short-term [20] and long-term tissue protection [21]. We also demonstrated that lithium prevented the loss of hippocampal neurogenesis after exposure to ionizing radiation and improved subsequent cognitive performance in mice [26].

All these studies demonstrate that lithium treatment soon after HI has both short- and long-term effects in reducing cell death and promoting repair. However, it is still unknown if delayed lithium administration, beyond the initial, major wave of cell death, has any long-term effects on neonatal brain injury. If the onset of treatment can be delayed, it would be much more attractive and relevant from a clinical point of view. Therefore, the potential long-term neuroprotective effects were investigated in this study where lithium administration was delayed 5 days after HI.

## Materials and Methods

### Hypoxia-ischemia

Male Wistar rat pups were purchased from Charles River and randomly assigned to HI and control groups, and unilateral HI was induced at postnatal day 8 (P8). Briefly, rat pups were anesthetized with isoflurane (5% for induction and 1.5%–2.0% for maintenance) in a 1∶1 mixture of nitrous oxide and oxygen. The left common carotid artery of each pup was isolated, and ligated with Prolene sutures (6.0) and cut between double ligatures. The wound was sutured and infiltrated with lidocaine for local analgesia after the surgical procedure. The duration of anesthesia and surgery was less than 5 min. The pups were returned to their dam and allowed to recover for one hour, and then placed in an incubator perfused with a humidified gas mixture (7.7% oxygen in nitrogen) at 36°C for 45 min. After hypoxic exposure, the pups were returned to their dams. Control animals were not subjected to surgery or hypoxia. All animal experimentation was approved by the Gothenburg Committee of the Swedish Animal Welfare Agency (application no. 90-2011).

### Lithium administration

Lithium chloride (Aldrich, St. Louis, MO, USA) was freshly prepared by dissolving in saline and injected intraperitoneally at a dose of 2 mmol/kg on the fifth day after HI (P13) (n = 21 for the vehicle-treated group and n = 23 for the lithium-treated group) or the same age of non-HI littermates (n = 15 for both vehicle- and lithium-treated control groups). Additional injections of 1 mmol/kg were administered at 24 h intervals for 14 consecutive days. Animals were sacrificed 12 weeks after HI (P91). Control rats were treated with vehicle alone, otherwise following the same protocol.

### Immunohistochemistry

The animals were deeply anesthetized with phenobarbital and perfused intracardially with 5% formaldehyde in 0.1 M phosphate buffer solution followed by immersion fixation in the same fixative at 4°C for 24 h. After dehydration with graded ethanol and xylene, the brains were paraffin-embedded, and cut into 5 µm coronal sections. Sections were deparaffinized in xylene and rehydrated in graded ethanol concentrations before staining. Antigen recovery was performed by heating the slides in 10 mM sodium citrate buffer, pH 6.0, for 10 min. Nonspecific binding was blocked in 4% horse, goat or donkey serum in phosphate-buffered saline for 30 min. Monoclonal mouse anti-MAP-2 (1∶1000 dilution, clone HM-2, Sigma, USA), rabbit anti-Iba1 (1∶500, Wako, Osaka, Japan), or rat anti-galectin-3 (5 mg/mL; eBioscience) or goat anti-doublecortin (DCX) (1∶200, sc-8066, Santa Cruz Biotechnology) antibody was applied to the tissue sample and incubated at 20°C for 60 min followed by biotinylated horse anti-mouse, goat anti-rabbit, or donkey anti-rat (1∶200, Vector Laboratories, Burlingame, CA, USA) secondary antibody for 1 h at 20°C. After blocking endogenous peroxidase activity with 3% H_2_O_2_ for 10 min, the sections were visualized with Vectastain ABC Elite (Vector Laboratories, USA) and 0.5 mg/mL 3, 3′-diaminobenzidine enhanced with ammonium nickel sulfate, beta-D glucose, ammonium chloride, and beta-glucose oxidase. For the double labeling of Iba1 and Galectin-3, antigen recovery was performed as above followed by incubation with the mixed antibodies in PBS at room temperature for 1 h. After washing, the sections were incubated with Alexa Fluor 488 donkey anti-rat IgG (H+L) and Alexa 555 donkey anti-rabbit IgG (H+L) secondary antibodies (1∶500, Jackson ImmunoResearch Laboratories) at 20°C for 1 h. After washing, the sections were mounted using Vectashield prolong mounting medium.

### Cell counting

The number of Iba1-positive cells was counted at 400× magnification in the border zone (surrounding the cavity) of the injured cortex by a person who did not have prior knowledge of the groups. Three sections were counted from each brain with an interval of 250 µm. The Iba1-positive cells were classified into ramified (resting/non-activated), hyper-ramified (reactive/intermediate), or un-ramified (activated/bushy or amoeboid) microglia according to morphological criteria [27]. The percentage of each type of microglia was calculated and compared between vehicle-treated and lithium-treated HI groups. DCX-labeled cells were counted in the subgranular zone of the contralateral hippocampus.

### Injury evaluation

Brain injury was evaluated using the total tissue loss volume and neuropathological scoring by a person who did not have prior knowledge of the groups. The MAP2-positive area in each section was measured in both hemispheres using Micro Image (Olympus, Japan). The volume was calculated from the MAP2-positive areas according to the Cavalieri principle, using the formula: V =  SA*T*P, where V is the total volume, SA is the sum of area measurements, T is the section thickness and P is the inverse of the sampling fraction. The total tissue loss was defined as the MAP2-positive volume in the contralateral hemisphere minus the MAP2-positive volume in the ipsilateral hemisphere. The neuropathological score for different brain regions was assessed. Briefly, the cortical injury was graded from 0 to 4 with 0 being no observable injury and 4 indicating confluent infarction. The injury in the hippocampus, striatum, and thalamus was assessed both with respect to hypotrophy (scored from 0 to 3) and injury/infarction (scored from 0 to 3), resulting in a total score of 22.

### Serum creatinine measurement

Blood samples were collected from the tail 5 weeks after HI (2 weeks after the end of the lithium treatment). Serum was isolated by centrifugation at 2,000×*g* for 15 min at 20°C and stored at −80°C. The serum creatinine level (mg/dL) was measured using the DetectX@ serum creatinine kit (Arbor Assays, Michigan, US. cat: KBO2-H1) according to the manufacturer's instructions.

### Multiplex cytokine/chemokine assay

Cytokine/chemokine levels were measured in serum. The samples were prepared according to the protocol from the manufacturer, and the levels of IL-1α, IL-1β, IL-6, IL-17, TNF-α, and IP10 (CXCL10) were measured simultaneously using the MILLIPLEX MAP rat cytokine magnetic bead panel kit (Millipore). The results are expressed as pg/mL.

### Open field

The motor activity patterns were analyzed with an open field and video tracking system 6 weeks after HI. Briefly, the animals were introduced to an unfamiliar open field arena and immediately videotaped at a sampling frequency of 12.5 Hz for 20 min. The open field arena (100 cm×100 cm) was videotaped from above using a CCD camera. The middle of the animal's body was defined as the point for tracking zone entries. The central zone was defined as a 30 cm×30 cm area in the center of the arena. The arena was made of gray Plexiglas and the floors were covered with sawdust. The walls were cleaned and the sawdust was moved around between trials. The recorded video was analyzed using the EthoVision 3.1 video-tracking software (Noldus Information Technologies, Netherlands). The generated variables were summarized into 5-minute bins. Data were analyzed with respect to the distance moved.

### Statistics

All data were expressed as the mean ± s.e.m. Student's *t*-test was used when comparing tissue loss. The Mann–Whitney U-test was used for comparing pathological scores, and the proportions of the different types of Iba1-positive cells. A two-way ANOVA was used when comparing the effects of lithium treatment and HI. Repeated measures ANOVA was used when comparing distance moved in the open field. The significance level was set to p<0.05.

## Results

### Lithium treatment reduced HI-induced brain injury

**Figure 1:**
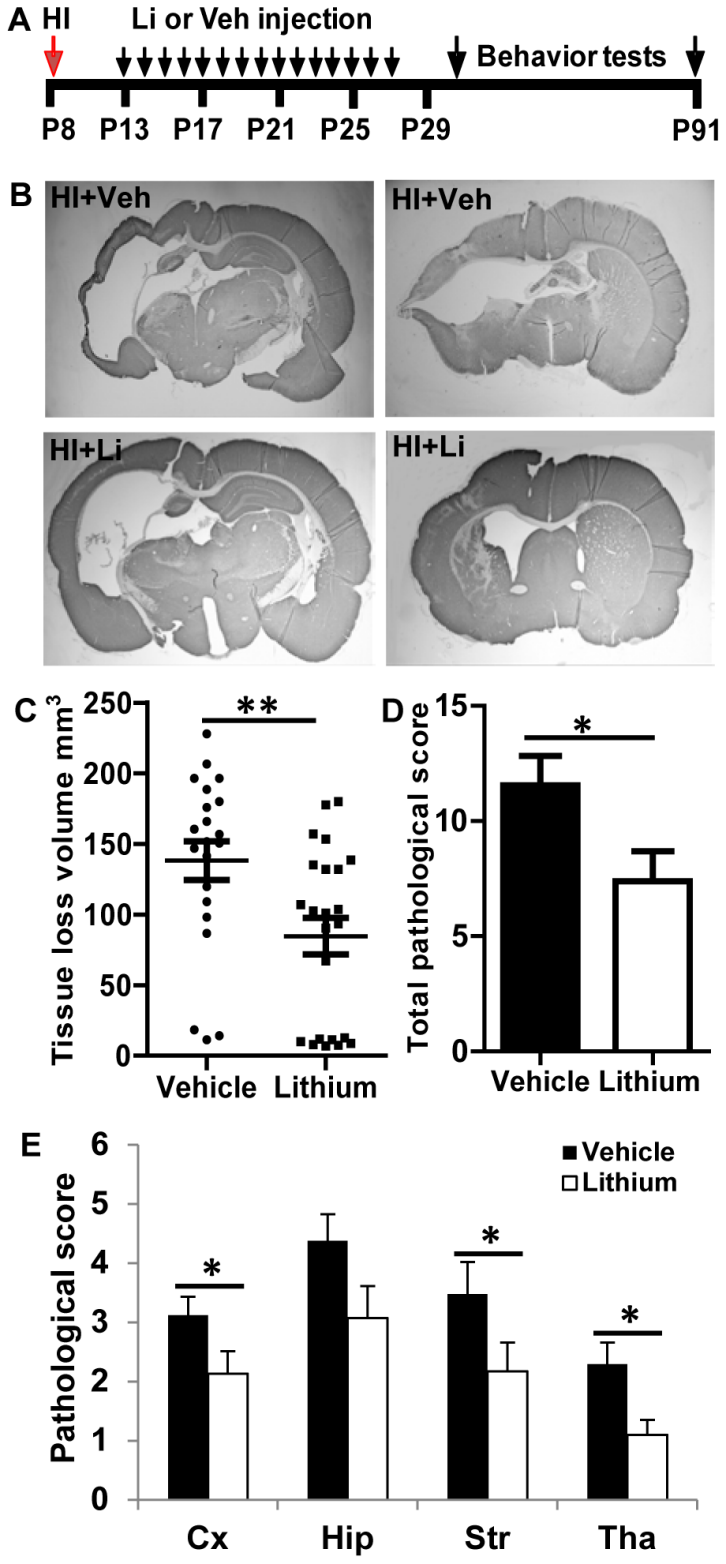
Lithium treatment reduced HI brain injury. **A.** The study design. **B.** Representative MAP2 staining from the dorsal hippocampus (left panels) and striatum (right panels) 12 weeks post-HI in vehicle-treated (upper panels) and lithium-treated rats (lower panels). **C.** The total volume of tissue loss 12 weeks after HI was reduced by 38.8% in lithium-treated mice (n = 23) compared with vehicle-treated mice (n = 21). **D.** The total pathological score. **E.** Neuropathological scores revealed less injury in the observed brain regions (except the hippocampus) after lithium treatment. Cx  =  cortex, Hip  =  hippocampus, Str  =  striatum, and Tha  =  thalamus; *p<0.05, **p<0.01.

Brain injury was evaluated 12 weeks after HI (Fig. 1A). In the vehicle-treated control rats, extensive brain injury was observed (Fig. 1B). Lithium treatment administered 5 days after HI and then for the next two weeks was effective at reducing the severity of this brain injury. The overall tissue loss was 276.5±27.4 mm^3^ in the vehicle-treated group and 169.3±25.9 mm^3^ in the lithium-treated group, corresponding to a 38.7% reduction in injury volume in response to the lithium treatment (p = 0.007) (Fig. 1C). Lithium treatment significantly reduced signs of injury in the cortex (p = 0.039), striatum (p = 0.040), and thalamus (p = 0.012), but not in the hippocampus (p = 0.078) (Fig. 1D–E).

### Lithium treatment normalized motor and anxiety-related activity

**Figure 2:**
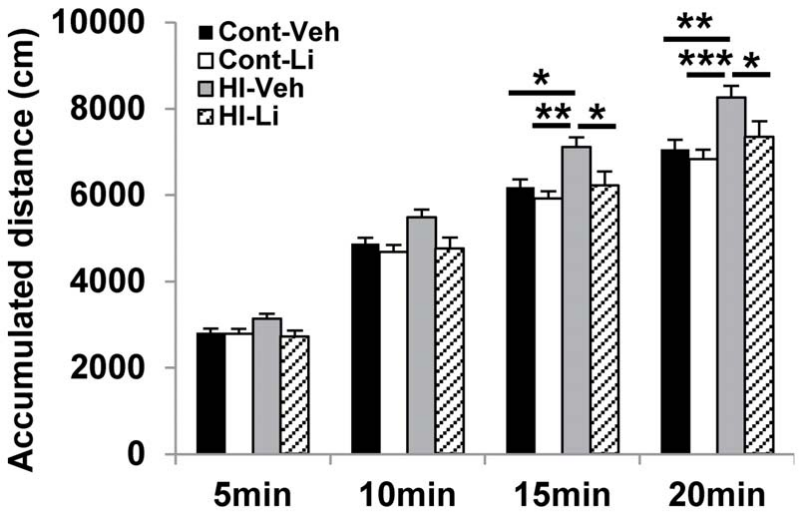
Open field. Accumulated distance moved. The HI rats treated with vehicle moved a longer distance during the 20 min analysis period (n = 13 for vehicle and n = 15 for lithium treated non-HI groups. n = 21 for vehicle and n = 23 for lithium treated HI groups). *p<0.05, **p<0.01.

As measured by the distance moved in the open field test, there was no difference in motor activity between the vehicle-treated and lithium-treated non-HI control groups. The rats subjected to HI at P9 showed increased motor activity, and subsequent lithium treatment normalized this (Fig. 2).

### Lithium treatment promoted neurogenesis

**Figure 3:**
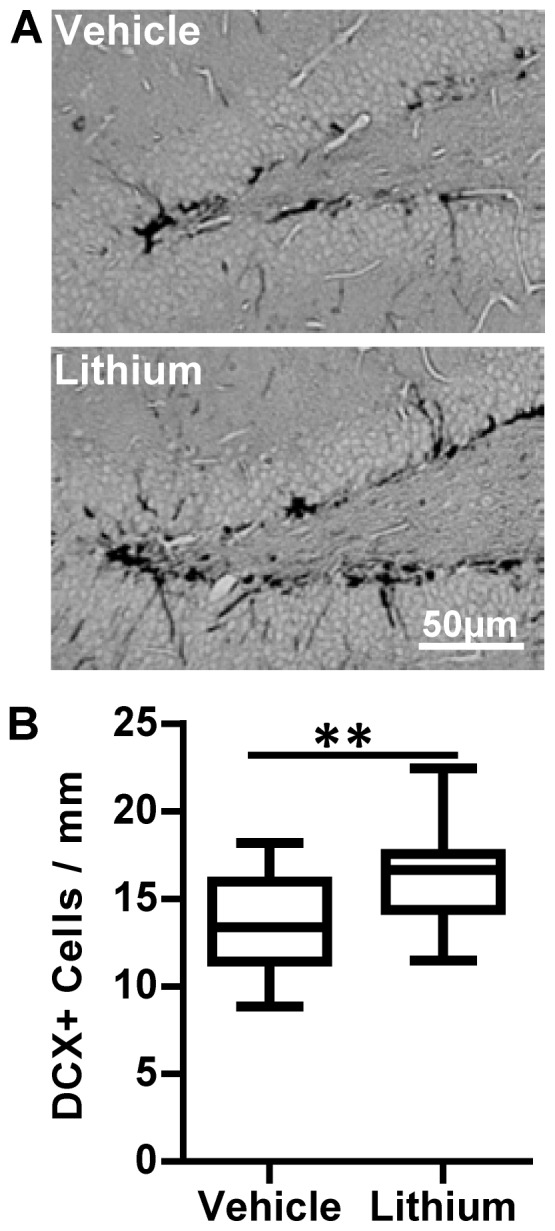
Neurogenesis in the dentate gyrus. **A.** Representative DCX staining in the contralateral dentate gyrus 12 weeks post-HI in vehicle-treated (upper panel) and lithium-treated (lower panel) rats, reflecting hippocampal neurogenesis. **B.** The number of DCX-positive cells was not significantly different between vehicle- and lithium-treated control groups (n = 13 for vehicle and n = 15 for lithium treated groups). The number of DCX-positive cells increased after the insult and was 21.3% higher in the lithium-treated group than in the vehicle-treated group (n = 21 for vehicle n = 13 for lithium treated group). **p<0.001.

Neurogenesis, as judged by the number of doublecortin (DCX)-positive cells in the dentate gyrus of the hippocampus, was evaluated in the contralateral hemisphere because the ipsilateral hippocampus was often too injured to be evaluated properly. The number of DCX-positive cells increased after hypoxia (the contralateral hemisphere is subjected to hypoxia, but not ischemia) and DCX counts revealed 13.6±0.6 cells/mm in the vehicle-treated group vs. 16.9±0.6 cells/mm in the lithium treatment group 12 weeks after HI (p = 0.0004) (Fig. 3).

### Lithium treatment reduced persistent microglia activation and inflammation after HI

**Figure 4:**
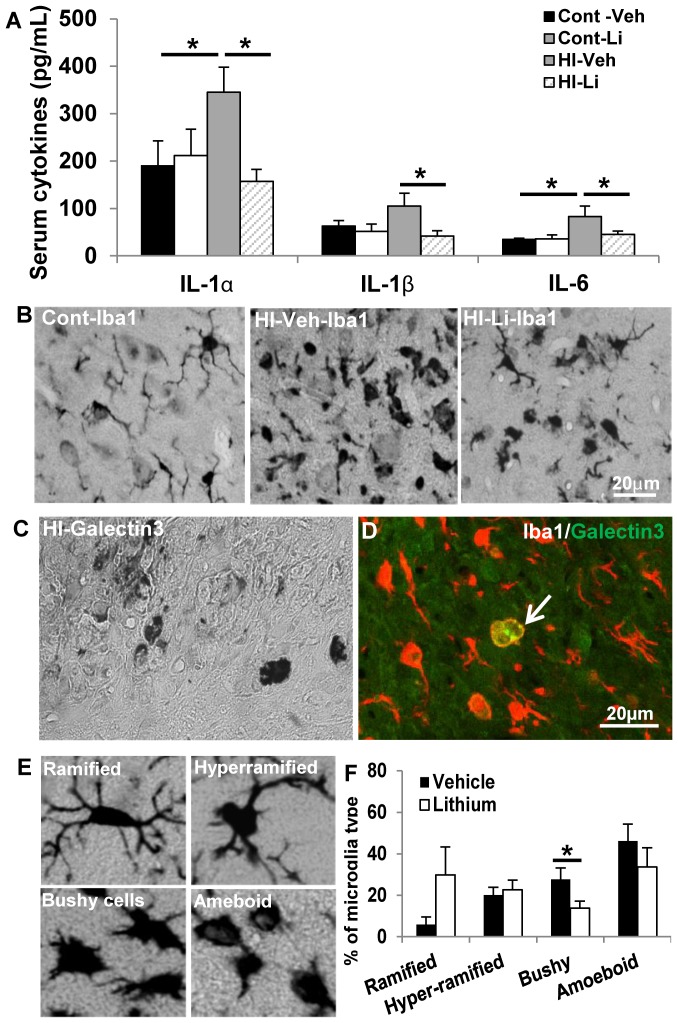
Serum cytokines and microglia activation. **A.** The serum levels of IL-1α, IL-1β, and IL-6 were increased after HI, and lithium treatment normalized these levels (n = 5/group). **B.** Representative Iba-1 immunostaining in the cortex of a normal control rat (left panel), an HI-exposed rat after vehicle treatment (middle panel), and an HI-exposed rat after lithium treatment (right panel) 12 weeks after HI. **C.** Representative galectin-3 staining in the border zone of the cortex 12 weeks after HI. **D.** Double labeling of Iba1 (red) and galectin-3 (green) in the border zone of the cortex 12 weeks after HI demonstrating that very few Iba1-positive cells were also galectin-3-positive. **E.** Morphology of Iba1-positive cells indicating ramified (surveillance microglia), hyper-ramified (intermediate) microglia, and un-ramified (bushy or amoeboid) microglia. **F.** Percentage of Iba1-positive cells according to morphology in the border zone of the cortical injury after HI (n = 10/group). Scale bar  = 20 µm. *p<0.05.

Serum cytokine levels were still increased in the vehicle-treated group as late as 5 weeks after HI. There was no difference between the vehicle-treated and lithium-treated controls. This increase was more pronounced for IL-1α, IL-1β, and IL-6, and lithium treatment prevented this HI-induced increase completely (Fig. 4A). Microglia, as indicated by Iba1 labeling, were scattered throughout the normal brain and became un-ramified (bushy or amoeboid) or activated in the cortex after HI, as assessed by morphological analysis (Fig. 4B). Pathologically activated microglial cells, as indicated by amoeboid morphology and galectin-3 labeling [21], were still detectable in the injury area 12 weeks after HI (Fig. 4C, 4D). The Iba1-labeled microglial cells were classified into ramified (resting), hyper-ramified (reactive or intermediate), un-ramified (bushy or amoeboid) or activated microglia based on their morphology (Fig. 4E). Most of the microglial cells in the border zone of the injury area (surrounding the cavity) were un-ramified even 12 weeks after HI (Fig. 4F). The percentage of un-ramified (bushy and amoeboid combined) Iba1-labeled cells was 73.9% 12 weeks after HI, and this was reduced to 48% in the lithium-treated group, out of which bushy microglia cells alone were reduced more than 50% (Fig. 4F).

### The effect of lithium on body and organ weights

**Figure 5:**
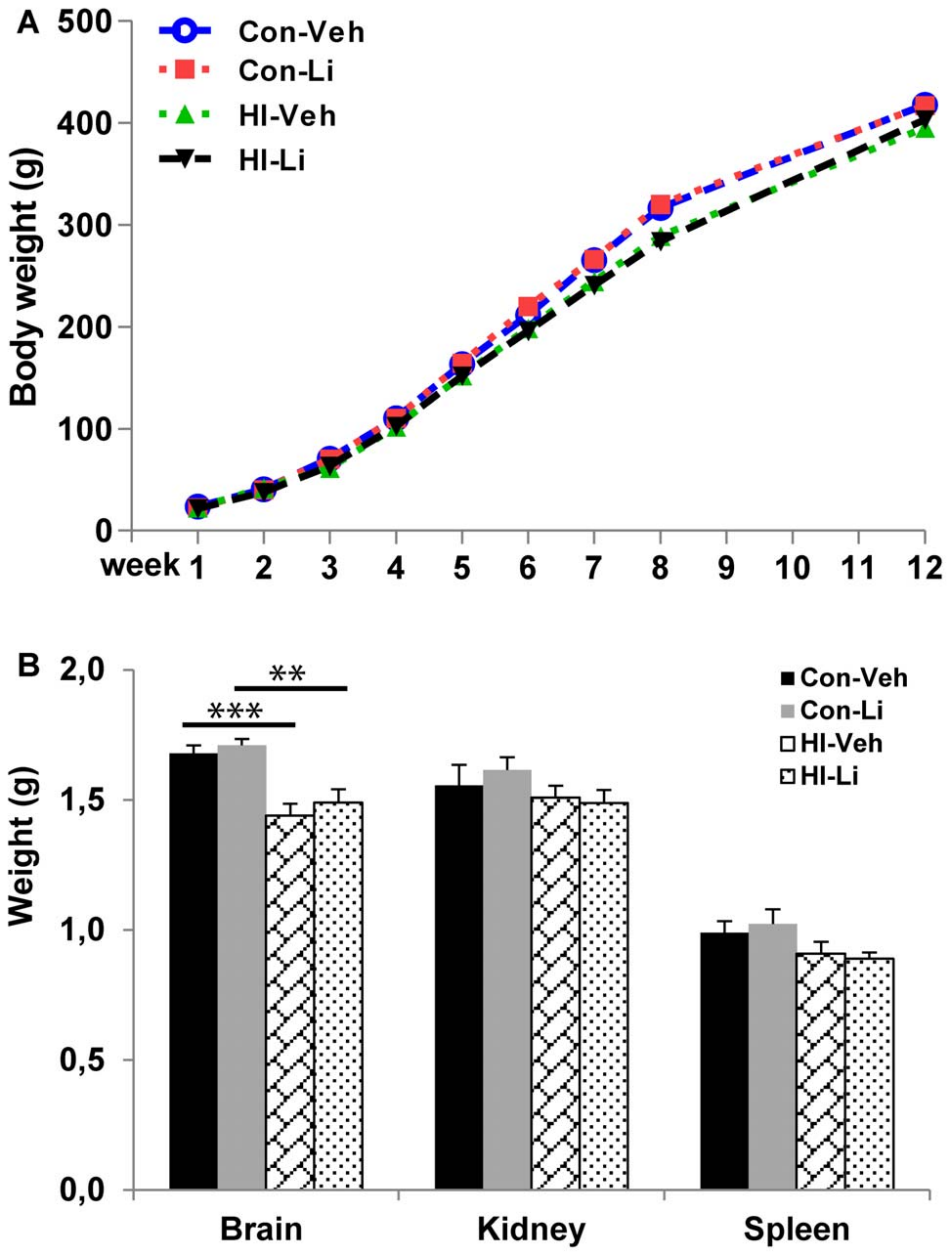
Body and organ weight. **A.** HI rats had a slightly slower body weight gain during weeks 6 to 8 after birth. Lithium treatment had no effect on body weight gain, neither in control rats nor after HI. **B.** The brain weight was lower after HI compared to the non-HI controls. The weights of the kidneys and spleens were not significantly different among the groups (n = 13 for vehicle and n = 15 for lithium treated non-HI groups. n = 21 for vehicle and n = 23 for lithium treated HI groups). **p<0.01, ***p<0.001.

The rats were weighed every day for 3 weeks after HI and then once every week. From 5 to 8 weeks the body weight gain was slightly lower in HI rats compared to non-HI control rats. There was no difference between vehicle-treated and lithium-treated rats, neither in the non-HI control group, nor in the HI group, demonstrating that the lithium treatment had no effect on body weight gain (Fig. 5A). The brain weight was lower in the rats subjected to HI when compared to the controls, but no difference was observed between vehicle-treated and lithium-treated groups. The kidney and spleen weights tended to be lower after the HI insult, but no significant differences were observed among the groups (Fig. 5B). Serum creatinine levels were not different between the vehicle-treated and lithium-treated animals 5 weeks after HI (0.139±0.035 pg/mL in the vehicle-treated group *vs.* 0.119±0.021 pg/mL in the lithium-treated group) (n = 9/group).

## Discussion

Despite significant progress in obstetric and neonatal care, perinatal HI brain injury is still one of the leading causes of neonatal mortality and morbidity [28]. Considerable effort has been devoted to developing novel therapeutic strategies based on the understanding of the pathophysiology of HI-induced brain injury [4]. However, only early application of hypothermia or administration of recombinant human erythropoietin has demonstrated clinical effectiveness in full-term infants suffering from brain injury [6], [15]. The current therapies for most neonatal brain injuries are still supportive and seek to maintain stable physiological parameters, and there is still no accepted therapeutic strategy for preventing late-phase cell death and chronic brain injury [5]. Extending the results of our previous studies, we have now demonstrated for the first time that delayed lithium treatment, with an onset beyond the initial wave of cell death, can reduce neonatal HI brain injury.

Lithium is an established drug for use in the treatment of bipolar disorder. However, recent studies have suggested that lithium also has neuroprotective properties and can reduce the severity of brain injury and subsequent functional deficits [20]–[22], [26], [29]–[31]. The cellular mechanisms underlying lithium's neuroprotective activity include the prevention of apoptotic cell death [20] and stimulation of neurogenesis and cell migration [21], [26]. We previously found that lithium treatment reduced apoptosis, autophagy, and brain injury in the neonatal rat brains 3 days after HI [20], but the protection 7 weeks after HI was even more profound (69% reduction of tissue loss after 7 weeks, compared with 43% after 3 days) [20]. This indicated that the long-lasting effects on continuous degeneration, stem cell proliferation, and possibly inflammation, might provide additional benefits. In this study, with lithium treatment delayed 5 days, the reduction in brain injury volume was 38.7%, i.e. much lower than the reduction seen after immediate lithium treatment (69%) [21]. This is most likely explained by early lithium administration inhibiting the massive apoptotic cell death occurring during the first days, peaking 1–2 days after HI. This is supported also by the pronounced protection observed after caspase inhibition or in the hypomorphic AIF mutation seen in harlequin mice [32]. Given the very low levels of detectable cell death beyond 3 days after HI, it is remarkable that lithium treatment with an onset as late as 5 days after HI reduced tissue loss by almost 40%. These findings demonstrate the importance of late-occurring degenerative events, and also inspire hope that even if an intervention is introduced as late as several days after an injury, it may confer substantial clinical benefit. Overall, neuroprotective strategies should target both the early and late degenerative events to maximize the benefit and improve functional outcome.

Neonatal HI induces a cerebral inflammation that includes an acute response and a prolonged inflammatory response, that is characterized by the production of cytokines, activation of resident glial cells, induction of leukocyte infiltration into the brain [11], [21]. Resident microglial cells are the main component of the innate immune system and play a key role in the phagocytosis of cell debris to maintain tissue homeostasis and support tissue repair. Microglial cells are among the first to become activated after HI, and activated microglia upregulate the expression of cytokines/chemokines that attract peripheral macrophages to the site of the injury [33]. Cytokines serve as important mediators of neuronal cell death [34], and targeting late-stage neuroinflammatory mechanisms might be a promising avenue for therapeutic interventions [35]. Attenuation of inflammation and microglial activation has been shown to confer neuroprotection in neonatal HI brain injury [21], [34], and we have previously demonstrated that increased microglia formation after HI in neonatal and juvenile mice, both in the hippocampus [36] as well as in the cortex and striatum [37], is concurrent with increased expression of the pro-inflammatory markers CCL2 (MCP1) and IL-18 [36], [37]. In this study, we found that serum cytokines were still elevated 5 weeks after HI and that galectin-3-positive microglia were located only in the injured areas, thereby serving as a marker of pathologically activated microglia that distinguished them from normal, ramified microglia [38]. The underlying mechanisms correlating delayed lithium treatment and reduced neuroinflammation remain to be elucidated, for example whether reduced inflammation is merely secondary to the lesser injury or if lithium may have a direct effect on microglia and/or other components of the immune system. This is essential in order to design treatments that can be safely administered over an extended period of time after the initial brain injury.

In summary, our study has demonstrated that lithium treatment initiated as late as after the major wave of cell death has occurred could reduce tissue loss by almost 40%, normalize motor and anxiety behavior, and also normalize the apparently chronic inflammatory response observed. No adverse effects were observed with lithium treatment. Lithium has been used in the treatment of bipolar disorder, also in children [39], but a safety evaluation is needed before lithium administration can be recommended as a treatment for neonatal brain injury. Nevertheless, these results open a new avenue for future development of clinical treatment strategies for neonatal brain injury, even well beyond the acute phase.
